# Supplementary material for: The association between smoking and cervical human papillomavirus infection among women from indigenous communities in western Botswana
Source: PLoS One. 2024 Jun 7;19(6):e0302153. doi: 10.1371/journal.pone.0302153 (PMC11161041; doi:10.1371/journal.pone.0302153)
Supplement: S1 Checklist — (DOC) [file pone.0302153.s001.doc]

STROBE checklist of items that should be included in reports of cross-sectional case-control observational studies with detailed referencing of requirements to the text of the paper.

|  | | Item No | Recommendation |
| --- | --- | --- | --- |
| **Title and abstract** | | 1 | (*a*) Indicate the study’s design with a commonly used term in the title or the abstract  Cross-sectional study as stated in the Abstract on page 2 |
|  |  |  | (*b*) Provide in the abstract an informative and balanced summary of what was done and what was found  Provided in Abstract on page 2. |
| Introduction | | | |
| Background/rationale | | 2 | Explain the scientific background and rationale for the investigation being reported  Included in the Introduction on pages 4 and 5. |
| Objectives | | 3 | State specific objectives, including any prespecified hypotheses  Included in the Introduction on page 5. |
| Methods | | | |
| Study design | | 4 | Present key elements of study design early in the paper  Included in the Methods section on pages 5 and 6. |
| Setting | | 5 | Describe the setting, locations, and relevant dates, including periods of recruitment, exposure, follow-up, and data collection  Included in the Methods on pages 5, 6 and 7 |
| Participants | | 6 | (*a*) *Cohort study*—Give the eligibility criteria, and the sources and methods of selection of participants. Describe methods of follow-up  *Case-control study*—Give the eligibility criteria, and the sources and methods of case ascertainment and control selection. Give the rationale for the choice of cases and controls  *Cross-sectional study*—Give the eligibility criteria, and the sources and methods of selection of participants  Included in the Methods section on page 6. |
|  |  |  | (*b*) *Cohort study*—For matched studies, give matching criteria and number of exposed and unexposed  *Case-control study*—For matched studies, give matching criteria and the number of controls per case  Not applicable. |
| Variables | | 7 | Clearly define all outcomes, exposures, predictors, potential confounders, and effect modifiers. Give diagnostic criteria, if applicable  Included in the Methods section on page 6. |
| Data sources/ measurement | | 8* | For each variable of interest, give sources of data and details of methods of assessment (measurement). Describe comparability of assessment methods if there is more than one group  Included in the Methods section on page 6. |
| Bias | | 9 | Describe any efforts to address potential sources of bias  Addressed in the context of discussion of limitations on page 17 |
| Study size | | 10 | Explain how the study size was arrived at  Included in the Methods section on page 5 and 6 |
| Quantitative variables | | 11 | Explain how quantitative variables were handled in the analyses. If applicable, describe which groupings were chosen and why  Included in the Methods under data management/analysis on pages 7 and 8. |
| Statistical methods | | 12 | (*a*) Describe all statistical methods, including those used to control for confounding  Included in the Methods section on pages 7 and 8. |
|  |  |  | (*b*) Describe any methods used to examine subgroups and interactions  No subgroup analyses performed. |
|  |  |  | (*c*) Explain how missing data were addressed  N/A. |
|  |  |  | (*d*) *Cohort study*—If applicable, explain how loss to follow-up was addressed  *Case-control study*—If applicable, explain how matching of cases and controls was addressed  *Cross-sectional study*—If applicable, describe analytical methods taking account of sampling strategy  Included in the Methods section on page 6. |
|  |  |  | (*e*) Describe any sensitivity analyses  Not applicable. |
| Results | | | |
| Participants | 13* | (a) Report numbers of individuals at each stage of study—eg numbers potentially eligible, examined for eligibility, confirmed eligible, included in the study, completing follow-up, and analysed  Included in the Results on page 8. | |
|  |  | (b) Give reasons for non-participation at each stage  Not applicable. | |
|  |  | (c) Consider use of a flow diagram  Not required. | |
| Descriptive data | 14* | (a) Give characteristics of study participants (eg demographic, clinical, social) and information on exposures and potential confounders  Included in the Results on pages 8 and 9. | |
|  |  | (b) Indicate number of participants with missing data for each variable of interest  Included in the Results on page 9. | |
|  |  | (c) *Cohort study*—Summarise follow-up time (eg, average and total amount)  Not applicable. | |
| Outcome data | 15* | *Cohort study*—Report numbers of outcome events or summary measures over time | |
|  |  | *Case-control study—*Report numbers in each exposure category, or summary measures of exposure | |
|  |  | *Cross-sectional study—*Report numbers of outcome events or summary measures  Included in the Results on pages 8 and 9 | |
| Main results | 16 | (*a*) Give unadjusted estimates and, if applicable, confounder-adjusted estimates and their precision (eg, 95% confidence interval). Make clear which confounders were adjusted for and why they were included  Included in the Results on pages 8 and 9 | |
|  |  | (*b*) Report category boundaries when continuous variables were categorized  Included in the Results on pages 8 and 9 | |
|  |  | (*c*) If relevant, consider translating estimates of relative risk into absolute risk for a meaningful time period. Not applicable. | |
| Other analyses | 17 | Report other analyses done—eg analyses of subgroups and interactions, and sensitivity analyses  Not applicable. | |
| Discussion | | | |
| Key results | 18 | Summarise key results with reference to study objectives  Included in the Discussion on page 14. | |
| Limitations | 19 | Discuss limitations of the study, taking into account sources of potential bias or imprecision. Discuss both direction and magnitude of any potential bias  Included in the Discussion on page 17. | |
| Interpretation | 20 | Give a cautious overall interpretation of results considering objectives, limitations, multiplicity of analyses, results from similar studies, and other relevant evidence  Included in the Discussion on pages 14 to 18. | |
| Generalisability | 21 | Discuss the generalisability (external validity) of the study results  Included in the Discussion on page 17. | |
| Other information | | | |
| Funding | 22 | Give the source of funding and the role of the funders for the present study and, if applicable, for the original study on which the present article is based  Provided as disclosure statement. | |
