# Supplementary material for: The association between smoking and cervical human papillomavirus infection among women from indigenous communities in western Botswana
Source: PLoS One. 2024 Jun 7;19(6):e0302153. doi: 10.1371/journal.pone.0302153 (PMC11161041; doi:10.1371/journal.pone.0302153)
Supplement: S1 Table — (DOCX) [file pone.0302153.s002.docx]

**Supplementary Table:** Bootstrap post-hoc analysis;

**Multivariable multilevel logistic regression analysis of the association between HPV DNA detection and individual/community level characteristics of women from indigenous communities in western Botswana**
