# Supplementary material for: The association between smoking and cervical human papillomavirus infection among women from indigenous communities in western Botswana
Source: PLoS One. 2024 Jun 7;19(6):e0302153. doi: 10.1371/journal.pone.0302153 (PMC11161041; doi:10.1371/journal.pone.0302153)
Supplement: Attachment — Submitted filename: Responses to reviewers_smoking and HPV_PLOS One.docx [file pone.0302153.s006.docx]

We are grateful for the valuable comments received from reviewers of our manuscript and believe this has further strengthened our manuscript. Below are our responses (in black text) to the reviewers’ comments highlighted in blue text:

Reviewer #1: Thank you for the opportunity to revise this manuscript. I think it has a great potential to advance our knowledge about the links between HPV and undesirable outcomes. A great strength of the article refers to its unique sample. Nonetheless, I would suggest the following:
- Follow the last guidelines from the STROBE statement, attaching the file and properly updating the text.
- The introduction fails to provide more details about HPV prevalence in the region in comparison to other parts of the world.

We have updated the introduction to provide more details about HPV prevalence in the region in comparison to other part of the world. [Line 88-99] in the revised manuscript. The STROBE statement form has been completed and supplied.

- The mechanisms underlying the association between HPV-related cervical diseases and smoking must be explored.

We have updated the introduction to further outline the mechanisms underlying the association between HPV-related diseases and smoking. [Line 94-101]

- The last paragraph of the introduction could include references when citing the global north. Moreover, hypotheses should be followed by references.

We have now provided references related to the statement about the disproportionate amount of literature exploring the HPV/smoking intersection in the global north. [Line 118]

- Inclusion and exclusion criteria must be explicated, as well as potential loss of participants (this is also clearly required by the STROBE statement).

We have revised the write up on our inclusion/exclusion criteria as recommended and in line with the STROBE statement.

- Data analysis plan should be supported by references. As the sample size was quite limited, have you considered other methods (i.e., bootstrapping)?

We have now provided supporting references for the data analysis plan. [Line 182, 195-196].

We considered bootstrapping as a technique to account for the limited sample size. The multilevel multivariable logistic regression analysis was repeated with bootstrapping to examine the relationship between smoking and HPV infection. We sampled with replacement, 350 replications (representing a doubling of the actual sample size of the study). The results from the bootstrapping analysis (shown below) were comparable to the initial analysis and did not change the conclusions (i.e. qualitatively, smokers were twice as likely to have cervical high-risk HPV infection detected compared to non-smokers).

- What was the achieved power?

The sample size for the study was based on the primary objective of determining the prevalence of high-risk HPV among women from indigenous communities in Botswana. The statistical power was thus set at 80% to estimate the sample size. Post-hoc power calculations for evaluating the secondary aim (determining the association between smoking and high-risk HPV infection) revealed that we had 93.8% power to detect a difference in the odds of high-risk HPV infection between smokers and non-smokers in the sample.

- In Table 1, why no include chi-squared comparisons or other relevant methods?

We have included a column in Table 1 to capture p-values related to the comparison carried out across the rows.

- Table 2 is quite confusing. Why haven’t you presented crude OR?

We have added crude OR associated with the univariate analysis.

- The discussion is a bit limited. In other words, if focused mostly on comparisons with other studies. I have missed more in-depth discussion about the tentative explanations for the results found in the study, including social determinants of health, health policies in the region, access to gynecological care, and others.

The discussion section has been critically reviewed in view of the reviewers’ comments. The discussion has been expanded to provide more depth of the authors’ explanation of the results.

- Can you upload you full questionnaire?

The questionnaire is provided.

Reviewer #2: The authors presented a study to investigate the influence of smoking on HR-HPV infections and this correlation with the risk of cervical cancer. The title summarises the content of the work appropriately. The abstract is satisfactory and the table is clear and interesting.
Although the manuscript can be considered already of high quality, I would suggest taking into account the following minor recommendations:
- I suggest another round of language revision, in order to correct a few typos and improve readability.

The paper has been revised for language and identified typographical errors have been corrected.

- Inclusion/exclusion criteria should be better clarified by extending their description.

We have revised the write up on our inclusion/exclusion criteria as recommended and in line with the STROBE statement.

- I find it interesting to include a reference to the long-term outcomes of patients with cervical cancer treated with a minimally-invasive approach compared to the "classic" open approach (see PMID: 37149905).

The reviewer’s recommendation for including this reference in acknowledged. On careful review of the suggested publication, it is apparent that the suggested publication’s focus is on treatment outcomes. Although treatment outcomes for early stage cervical cancer are an important consideration, our study was focused on screening/diagnosis rather than treatment. An important future direction from this work will include an assessment of treatment outcomes in this population. We have included the suggested reference to support recommendations for future work evaluating treatment outcomes relative to the risk associated with smoking and HPV infection in our setting.

- I think that the discussion could be studied in depth. In fact, some aspects should be highlighted. In particular, the importance of cervical cancer screening and vaccination should be improved by analyzing the obstetrical outcomes and the positive margins persistence in patients. This could emphasize the importance of screening. I suggest these articles to get deeper in the topic : PMID: 33140628.

We note the reviewer’s suggestion for a discussion that includes cervical cancer screening, vaccination, obstetrical outcomes and positive margins (following surgical treatment). We agree that such a discussion would provide a more comprehensive overview of cervical HPV related disease continuum of care. However, given that our scope was limited to screening/diagnosis of cervical HPV related disease in the context of smoking as a risk factor among an indigenous population, we have not included all the issues surrounding the subject matter. We believe this approach keeps the discussion within the bounds of our stated aim of the study.

- The authors have not adequately highlighted the strengths of their study. I suggest better specifying these points.

We have expanded the discussion on the strengths of the study.

-What are the implications of these findings for clinical practice and/or further research?

We have revised the discussion section to include implications of the study findings for clinical practice and further research.

Reviewer #3: I read with great interest the Manuscript titled “The Association Between Smoking and Cervical Human Papillomavirus Infection Among Women From Indigenous Communities in Western Botswana." which falls within the aim of the Journal.

Although the manuscript can be considered already of good quality, I would suggest following recommendations:
- I suggest round of language revision, in order to correct few typos and improve readability;

The paper has been revised for language and identified typographical errors have been corrected.

- Considering topic and results if this study, I suggest that authors to add a reference to current evidence about crucial role of HPV infection persistence, also after treatment of cervical lesions. I would be glad if the authors discuss this important point PMID: 37401466 and 37747763.

We thank the reviewer for pointing out these important and recently published data. We found these relevant to the subject of our study and have incorporated them in our discussion.

Because of these reasons, the article should be revised and completed. Considering all these points, I think it could be of interest to the readers and, in my opinion, it deserves the priority to be published after minor revisions.

Reviewer #4: Sample size is small. Association could also have been studied with Chi-Square test and significance levels, along with the Models. The study is a simple cross sectional survey. To make it suitable for publication in a good journal, either increase the sample size or add other extensive variables too.

We appreciate the reviewer’s comment concerning the small sample size of our study. Indeed this is a limitation of our analysis and this is acknowledged in the discussion. Despite the limited sample size, we believe these data are critical in adding to what is known about the association between smoking and HPV infection in the context of a marginalised indigenous population.
