# Supplementary material for: The association between smoking and cervical human papillomavirus infection among women from indigenous communities in western Botswana
Source: PLoS One. 2024 Jun 7;19(6):e0302153. doi: 10.1371/journal.pone.0302153 (PMC11161041; doi:10.1371/journal.pone.0302153)
Supplement: Attachment — Submitted filename: Responses to the reviewers_2.docx [file pone.0302153.s007.docx]

Responses to the reviewers round 2

We are grateful for further comments received from the reviewers and have addressed these in our revised manuscript. Below are our responses (in black text) to the reviewers’ comments highlighted in blue text:

Reviewer #1: I am happy with the updated manuscript. The only think that is still missing is the bootstrap method. Usually, we need 10,000 resamplings. Authors just doubled their sample size. I would suggest another look at this in face of previous studies with comparable sample size.

We sampled with replacement, 10 000 replications as suggested. The results from the bootstrap analysis (shown below) were comparable to the initial analysis and did not change the conclusions (i.e. qualitatively, smokers were twice as likely to have cervical high-risk HPV infection detected compared to non-smokers). We have included a narration of this post hoc bootstrap analysis in the revised manuscript [line 340-342] and are present these results in a supplementary table.

Reviewer #4: The Chi-Square test has still not been used. It is just a cross -sectional survey and studies a basic known association. Statistical Analysis could have been stronger, and in detail.

We would like to confirm that the Chi-square test was used. The Chi-square test (or Fisher exact test where appropriate) was used to compare participants’ socio-economic and clinical characteristics in Table 1. We have highlighted this in the revised manuscript.[line 181-183]
